# The Impact of the SUPPORT Act on Treatment of Opioid Use Disorder

**DOI:** 10.1093/geroni/igaf122.3397

**Published:** 2025-12-31

**Authors:** Junjie Gai, Kanika Arora

**Affiliations:** University of Iowa, Iowa City, Iowa, United States; University of Iowa, Iowa City, Iowa, United States

## Abstract

On January 1, 2020, Medicare Part B began covering opioid use disorder (OUD) treatments, including medications and outpatient services, under the implementation of the Substance Use-Disorder Prevention that Promotes Opioid Recovery and Treatment for Patients and Communities (SUPPORT) Act of 2018. However, little is known about how this coverage expansion affects OUD treatment uptake among Medicare beneficiaries. This study examines whether the passage of the SUPPORT Act influenced the likelihood of medication treatment and treatment completion for OUD patients in Medicare compared to privately insured individuals. We applied a difference-in-differences and event study methodology to examine the causal impact of the SUPPORT Act among Medicare beneficiaries with OUD. We acquired datasets from 2015 to 2022 Treatment Episodes Datasets - Discharge. Our primary outcome is a binary variable (1/0) that indicates whether the use of opioid medications such as methadone or buprenorphine is part of the treatment. Other outcomes include outpatient and inpatient service, and treatment completion rate. All analyses are clustered at the state level and adjusted by covariates. After Medicare’s coverage in 2020, Medicare beneficiaries with OUD were 11.9 percentage points (pp) more likely (p < 0.05) to receive medication for OUD relative to the privately insured group. Notably, Medicare beneficiaries are more likely to receive non-intensive services, showing a 27.1 pp increase (p < 0.05). Our findings indicate a positive significant effect of Medicare’s expanded coverage on increasing access to medication treatment and outpatient services among older adults with OUD.

